# Isolation Housing Exacerbates Alzheimer’s Disease-Like Pathophysiology in Aged APP/PS1 Mice

**DOI:** 10.1093/ijnp/pyu116

**Published:** 2015-03-04

**Authors:** Huang Huang, Linmei Wang, Min Cao, Charles Marshall, Junying Gao, Na Xiao, Gang Hu, Ming Xiao

**Affiliations:** Jiangsu Key Laboratory of Neurodegeneration, Nanjing Medical University, Nanjing, Jiangsu, China (Drs Huang MD, Wang MD, Cao Ms, Gao Ms, N. Xiao Ms, Hu MD, PhD, and M. Xiao MD, PhD); Department of Rehabilitation Sciences, University of Kentucky Center for Excellence in Rural Health, Hazard, KY (Dr Marshall PhD).

**Keywords:** Alzheimer’s disease, β-amyloid, hippocampus, memory deficiency, social isolation, APP/PS1 mice

## Abstract

**Background:**

Alzheimer’s disease is a neurodegenerative disease characterized by gradual declines in social, cognitive, and emotional functions, leading to a loss of expected social behavior. Social isolation has been shown to have adverse effects on individual development and growth as well as health and aging. Previous experiments have shown that social isolation causes an early onset of Alzheimer’s disease-like phenotypes in young APP695/PS1-dE9 transgenic mice. However, the interactions between social isolation and Alzheimer’s disease still remain unknown.

**Methods:**

Seventeen-month-old male APP695/PS1-dE9 transgenic mice were either singly housed or continued group housing for 3 months. Then, Alzheimer’s disease-like pathophysiological changes were evaluated by using behavioral, biochemical, and pathological analyses.

**Results:**

Isolation housing further promoted cognitive dysfunction and Aβ plaque accumulation in the hippocampus of aged APP695/PS1-dE9 transgenic mice, associated with increased γ-secretase and decreased neprilysin expression. Furthermore, exacerbated hippocampal atrophy, synapse and myelin associated protein loss, and glial neuroinflammatory reactions were observed in the hippocampus of isolated aged APP695/PS1-dE9 transgenic mice.

**Conclusions:**

The results demonstrate that social isolation exacerbates Alzheimer’s disease-like pathophysiology in aged APP695/PS1-dE9 transgenic mice, highlighting the potential role of group life for delaying or counteracting the Alzheimer’s disease process.

## Introduction

Alzheimer’s disease (AD), the most common neurodegenerative disorder in the elderly, is reaching epidemic levels with tremendous social and financial burdens (Querfurth and Laferla, 2010). Unfortunately, the specific mechanisms causing AD remain unknown, and scientifically proven pharmacological treatment options are inadequate (Huang and Mucke, 2012). This urgent situation underscores the need for identifying crucial factors and/or mechanisms involved in accelerating the progression of AD and developing nonpharmacological interventions to slow the process of cognitive decline.

Social isolation (SI) refers to a complete, or near complete, lack of contact with people and society for members within a social species. This isolation has been proven to be harmful to individual development and growth as well as physical and mental health (Cacioppo et al., 2011). SI can be an issue for anyone, irrespective of their age. However, when compared with other age groups, the elderly are more vulnerable to suffer from SI because of declines in overall health, absent or uninvolved relatives or children, the abrupt end of daily work relationships after retirement, and/or economic struggles (Díez et al., 2014; Hand et al., 2014).

SI in elderly is associated with increased mortality risk and onset of several neuropsychological disorders, including schizophrenia (Jiang et al., 2013b), bipolar disorder (Miklowitz, 2011; Gilman et al., 2014), and AD (Dong and Csernansky, 2009). SI influences the onset and development of a mouse AD model via enhancing activity of β and γ-secretases and aberrant activation of p25/Cdk5, which plays an important role in the production of Aβ peptide and phosphorylation of tau, respectively (Hsiao et al., 2011). Recently, several studies have indicated that SI increases oxidative stress and inflammatory reaction (Powell et al., 2013) and inhibits antiinflammatory responses (Azzinnari et al., 2014), synaptic plasticity (Djordjevic et al., 2009), and myelination (Liu et al., 2012a), all of which are involved in the pathogenesis of AD.

Although SI may contribute to the onset of AD, the added effect on the disease progression remains unclear. Furthermore, AD patients, especially those in the late stages, are more likely to suffer from SI due to cognitive and emotional impairment and loss of communication ability as well as potential neglect of communication by family members or attendants (Tanzi and Bertram, 2005; Peña-Longobardo et al., 2014; Zucchella et al., 2014). Thus, it is necessary to determine whether SI exacerbates pathology of late-stage AD. A better understanding of the interplay between the 2 disease processes will help establish appropriate and effective interventions to delay or counteract the neurodegenerative progression of AD.

In the present study, we evaluated the effect of isolation housing on AD-like pathophysiology in aged APP695/PS1-dE9 transgenic (APP/PS1) mice. Our results demonstrate that 17-month-old APP/PS1 mice housed separately for 3 months displayed increased cognitive impairment, Aβ plaque burden, hippocampus atrophy, and reactive astrogliosis compared with group-housed controls.

## Materials and methods

### Animals and Experimental Design

Male APP/PS1 mice were used in this study. On postnatal day 28, weaned, male APP/PS1 mice were separated from their female littermates and housed (4 mice per cage) in a temperature- and humidity-controlled facility on a 12-h-light/-dark cycle with food and water ad libitum. At 17 months old, one-half of the animals were randomly selected and singly housed; the other one-half continued group housing until 20 months old. All experiments were conducted in accordance with international standards on animal welfare and the guidelines of the Institute for Laboratory Animal Research of Nanjing Medical University. All efforts were made to minimize animal suffering and reduce the number of animals used.

### Open Field Test

After a 2-day acclimatization to the behavioral testing room, mice performed the open field test so we could measure exploration and anxiety-related behaviors (Chen et al., 2006). The open field box consisted of a square black Plexiglas box (60 cm×60 cm×25cm), with an outlined center area (30 cm×30cm). Each animal was placed in the middle of box, which served as a starting point, then allowed to move freely for 10 minutes within the box. The amount of time and distance traveled in the center area of the maze, number of entries into the center, and grooming numbers were measured. At the conclusion of the experiment period, defecation number was also counted.

### Y-Maze Test

One day after the open field test, the Y maze was performed to measure mouse short-term memory, as previously described (Wang et al., 2013). In brief, the Y maze was randomly assigned to 3 arms: novel arm (NA), starting arm (SA), and other arm (OA). The Y maze test contains two 5-minute stages with an interval of 2 hours. During the first stage, the NA was blocked by a black baffle, with mice entering the SA were allowed to move freely only between the SA and OA. During the second stage, the NA was opened and mice entering from SA could freely move throughout 3 arms. The percentage of time traveled in each arm, number of entries into each arms and travelling speed were analyzed.

### Morris Water Maze

The Morris water maze task was conducted to measure long-term learning and memory function as described previously (Liu et al., 2012b). Briefly, a black plastic pool with a diameter of 100cm and a height of 50cm was filled with water (22±2°C). Training was conducted for 7 consecutive days with 4 trials/d. During the first 2 days of testing, mice were trained with a visible platform. A cylindrical dark-colored platform with a diameter of 10cm was placed 0.5cm above the water surface and kept consistently within one of the quadrants. On day 3, hidden platform testing was performed, in which the platform was moved to the opposite quadrant and submerged 1cm below the surface of the water. The escape latency, swim distance and speed, and swim patterns were analyzed. A probe trial was conducted on day 8; the hidden platform was removed, and mice were placed in the pool and allowed to swim for 60 seconds. The percent of total time spent in each quadrant and the number of crossing where the platform had been previously located were calculated.

Mouse activity in the above behavioral apparatuses was collected by a digital video camera connected with a computer-controlled system (Beijing Sunny Instruments Co. Ltd). All tests were performed by 2 independent experimenters who were blind to the treatment schedule.

### Section Preparation

Subsequent to deep anesthesia, mice were transcardially perfused with 0.9% saline by perfusion pump (Cole-parmer) for 5 minutes, followed by 4% paraformaldehyde for 12 minutes. Brains were dissected in the mid-sagittal plane, postfixed overnight at 4°C, then dehydrated in a series of graded ethanol solutions and embedded in paraffin. Both half-brains were serially cut into 5-µm sagittal sections using a paraffin slicing machine (Leica RM2135, Nussloch, Germany). Sections from one half-brain were divided into 5 sets for HE staining and Thioflavin-S staining, while serial sections from the other half-brain were used for immunohistochemical stains.

### Quantitative Analysis of Hippocampal Volume and Aβ Plaque Load

The dorsal hippocampus stained with H&E, Thioflavin-S or 6 E10 was photographed at 100× magnification using a digital microscope (Leica Microsystems). Individual images were exported to Image-Pro Plus 6.0 Analysis System (Media Cybernetics Inc., San Francisco, CA). For quantitative analysis of hippocampal volume, the boundary of hippocampus and gray region that consists of pyramidal cell layer and granular cell layer were manually delineated. The total hippocampal area and gray matter area per section were measured. Total hippocampal (gray matter) volume per mouse was also calculated according to Cavalieri’s estimator (Pereda-Pérez et al., 2013), V=(S1+2S2+2S3+…+2Sn-1+Sn) µm^2^×5 µm×5. The hippocampal white matter volume was obtained by the total hippocampal volume subtracting the hippocampal gray matter volume. For quantitative analysis of Aβ plaque load, thioflavin-S positive signals were determined by standardized region of interest grayscale threshold analysis. The cross area of each plaque and total area of plaque coverage relative to the total hippocampus area were also measured.

### Immunohistochemistry

Immunohistochemical staining was performed as previously described (Xu et al., 2013). Briefly, after deparaffinization and rehydration, tissue sections were incubated with a primary antibody direct against glial fibrillary acidic protein (GFAP) (1:1000; Sigma-Aldrich), ionized calcium-binding adaptor molecule 1 (Iba-1) (1:500; Wako), double-cortin (DCX) (1:500; Abcam), or proliferating cell nuclear antigen (PCNA) (1:1000; Abcam) at 4°C overnight. Following incubation (1 hour 30 minutes) with biotinylated IgGs and ABC (1 hour 15 minutes), the reaction was visualized with DAB (Sigma-Aldrich). The mean integrated optical density (IOD/total area) was measured to assess the expression level of GFAP and Iba-1 in the entire hippocampus at 100× magnification using an Image-Pro Plus 6.0 Analysis System (Media Cybernetics Inc). The number of DCX-positive cells and PCNA-positive cells per section was also counted.

### Western Blot

Hippocampal tissues were homogenized and centrifuged at 4°C and 12000rpm for 15 minute. The samples were resolved on SDS-PAGE, transferred onto PVDF membranes using a Bio-Rad miniprotein-III wet transfer unit, then blocked with 5% skim milk dissolved in TBST (pH 7.5, 10mM Tris-HCl, 150mM NaCl, and 0.1% Tween 20) at room temperature for 1 hour. Membranes were probed at 4°C overnight with a primary antibody directed against Aβ_1–42_ (1:1000, Abcam), β-amyloid precursor protein (sAPPα) (1:100; Immuno-Biological Laboratories), disintegrin and metalloproteinase10 (1:1000; Millipore), β-site amyloid precursor protein–cleaving enzyme 1 (BACE1) (1:2000; Millipore), presenilin1 (PS1) (1:1000; Sigma-Aldrich), neprilysin (NEP) (1:1000; Millipore), insulin degrading enzyme (IDE) (1:1000; Abcam), 6E10 (1:1000; Covance), myelin basic protein (MBP) (1:200; Santa Cruz Biotechnology), synaptophysin (SYP) (1:1500; Millipore), neuronal class III β-tubulin (1:3000; Sigma-Aldrich), or β-tubulin (1:3000; Sigma-Aldrich). Horseradish peroxidase-conjugated secondary antibodies (Vector Laboratories, Burlingame, CA) were used, and bands were visualized using ECL plus detection system. β-Tubulin was used as an internal control for protein loading and transfer efficiency.

### Statistical Analysis

All data were expressed as means ± SEM. The effects of treatment, training day, and their interaction on the behavioral performance in the Morris water maz trainineg task were evaluated by overall ANOVA for repeated measures. Other data were analyzed by 2-tailed Student’s *t* test. A *P* value < .05 was considered statistically significant.

## Results

### Isolation Housing Decreased Learning and Memory of Aged APP/PS1 Mice

**Figure 1.**
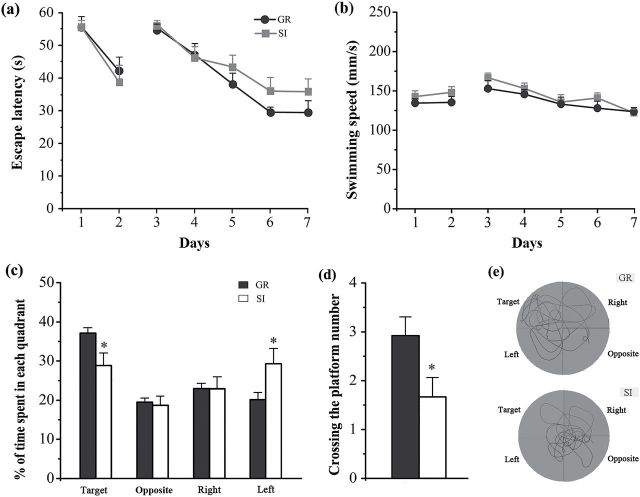
Morris water maze test. (a) The mean escape latency in the visible platform test (days 1–2) and hidden platform test (days 3–7). (b) Swimming speed. (c) Percentage of time spent in each quadrant in the probe test. (d) Crossing the platform number. (e) Tracings of the typical swim patterns in the probe test. Data represent means±SEM. * *P*<.05, socially isolated (SI) aged APP/PS1 mice (n=12) vs group (GR) controls (n=13).

The Morris water maze data showed that escape latency of isolated APP/PS1 mice was extended during hidden-platform acquisition training compared with group controls (group effect: F_1,115_=2.889, *P*=.049) (Figure 1a). However, the difference was not observed on visible platform testing (group effect: F_1,46_=0.188, *P*=.669) (Figure 1a). Isolated APP/PS1 mice swam faster than group mice in both visible platform testing and hidden-platform testing, but the difference was not statistically significant (group effect: F_1,46=_0.994, *P*=.329; F_1,115_=0.923, *P*=.470, respectively) (Figure 1b). These results suggest that isolation would specifically impair spatial learning ability but did not affect motor and/or visual ability. Additionally, isolated APP/PS1 mice displayed further spatial memory declines in the probe test, as revealed by decreased percentages of time spent in the target quadrant (*P*=.023) (Figure 1c) and the number of target quadrant crossing (*P*=.032) (Figure 1d). Swimming trial analysis showed that isolated APP/PS1 mice swam in each quadrant irregularly, but group controls had a preference for the target quadrant (Figure 1e). These results suggest that isolated APP/PS1 mice exhibited further deterioration of long-term memory.

**Figure 2.**
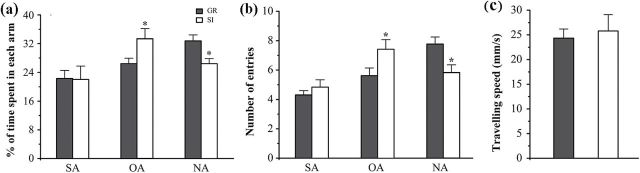
Y maze test. (a) Percentage of time spent in the novel arm (NA), starting arm (SA), and other arm (OA). (b) Number of arm entrances. (c) Travelling speed during the test. Data represent means ± SEM. * *P* < .05, socially isolated (SI) aged APP/PS1 mice (n=12) vs group (GR) controls (n=13).

Consistently, short-term memory declines were also more severe in isolated APP/PS1 mice during the Y maze test, as revealed by decreased percentages of time spent in the NA (*P*=.01) (Figure 2a) and number of NA entrances (*P*=.012) (Figure 2b). In addition, isolated mice moved slightly faster than group controls (*P*>.05) (Figure 2c), suggesting the occurrence of mild hyperactivity.

### SI Decreased Exploring Behaviors of Aged APP/PS1 Mice

**Figure 3.**
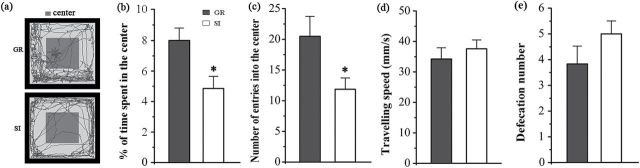
The open field test. (a) Tracing of mouse movement during the 10-minute test period. (b) Percentage of time spent in the center area. (c) Number of entries into the center area. (d) Travelling speed. (e) Defecation number during the test. Data represent means ± SEM. * *P* < .05, socially isolated (SI) aged APP/PS1 mice (n=12) vs group (GR) controls (n=13).

Results of the open field test suggest that isolated APP/PS1 mice showed a decline in exploratory activity compared with group controls (Figure 3a), as revealed by decreases in the time spent in the center area (*P=*.011) (Figure 3b) and the number of crosses into the center area (*P*=.029) (Figure 3c). In addition, isolated APP/PS1 mice exhibited mild anxiety-like behaviors, reflected by a tendency to increase movement speed (*P*=.484) (Figure 3d) and defecation number (*P*=.203) (Figure 3e).

### SI Increased Aβ Accumulation via Upregulation of PS1 and Downregulation of NEP in the Hippocampus of Aged APP/PS1 Mice

**Figure 4.**
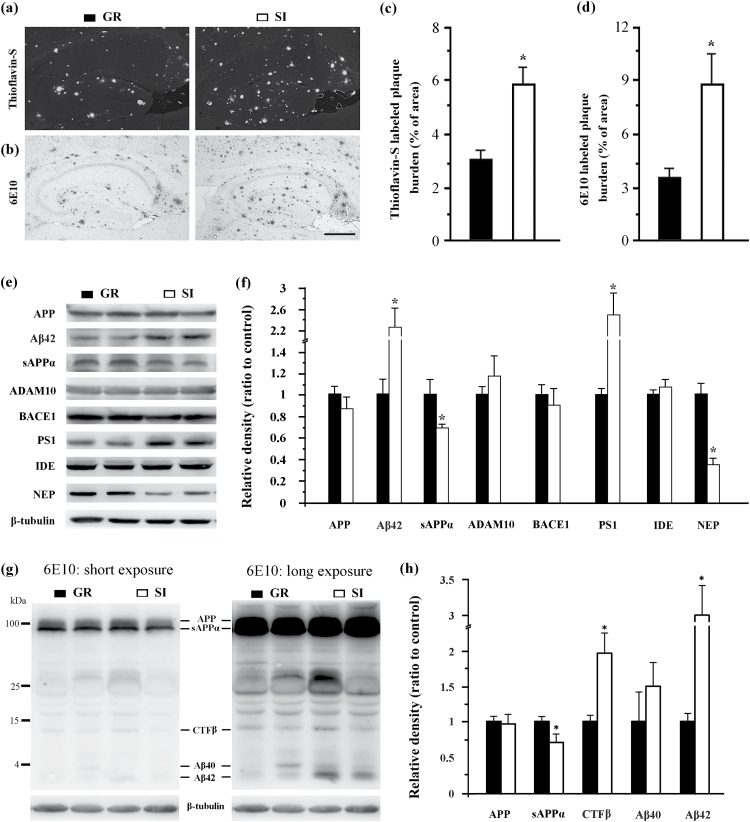
Analyses of amyloid metabolism-related indexes in the hippocampus. (a-b) Brain sections from isolated aged APP/PS1 mice and group controls were stained with Thioflavin-S (a) or immunostained with anti-6E10 antibody (b) to label plaques. Scale bar=500 μm. (c-d) Percentage of brain area occupied by Thioflavin-S (c) or 6E10 (d) labeled Aβ deposition in the hippocampus. (e-f) Western blotting and densitometry analysis of APP and its hydrolysis products, Aβ42 and sAPPα, and hydrolytic enzymes a disintegrin and metalloproteinase (ADAM) family protease ADAM10, β-site amyloid precursor protein-cleaving enzyme 1 (BACE1) and PS1 as well as Aβ-degrading enzymes IDE and NEP. (g-h) Western blotting using 6E10 and densitometry analysis showed expression levels of APP, sAPPa, CTFβ, Aβ40, and Aβ42. Data represent means±SEM from 5 mice per group for pathological analyses and from 3 mice per group in 3 independent Western blotting analysis experiments. *P < .05, socially isolated (SI) aged APP/PS1 mice vs group (GR) controls.

Isolation housing increased amyloid deposits in the hippocampus, visualized as Thioflavin-S positive dense core plaques (Figure 4a) and 6E10-immunopositive diffuse and mature plaques (Figure 4b). Quantification data revealed that the percentage area occupied by Thioflavin-S or 6E10 labeled plaques was higher in the hippocampus of aged APP/PS1 mice than in group controls (*P*=.013; *P*=.033, respectively; Figure 4c-d).

To further reveal the molecular mechanisms of SI effects on plaque pathogenesis, we examined the expression of proteins involved in Aβ synthesis and degradation using Western-blot analysis (Figure 4e-f). There was no difference in APP levels between isolated and group APP/PS1 mice (*P*=.211), suggesting that SI does not alter APP generation. However, SI altered APP hydrolysis in the hippocampus of isolated APP/PS1 mice, as revealed by prominent increases in the amyloidogenic peptide Aβ_1–42_ (*P*=.023), and mild decreases in the nonamyloidogenic peptide sAPPα compared with their group controls (*P*=.05). Consistent with altered hydrolysis products of APP, APP/PS1 isolated mice exhibited significantly higher levels of PS1 (γ-secretase) (*P*=.046), which is responsible for cleavage of APP C-terminal fragment CTFβ to create the Aβ peptide (Donmez et al., 2010). Expression levels of disintegrin and metalloproteinase10 (α-secretase) (*P*=.462) and β-site amyloid precursor protein–cleaving enzyme 1 (β-secretase) (*P*=.725) were not altered. These enzymes are required to cut APP to generate N-terminal soluble fragment sAPPα and C-terminal fragment CTFα, and sAPPβ and CTFβ, respectively (Obregon et al., 2012). Increased expression of CTFβ (*P*=.029) and Aβ42 (*P*=.025) and decreased expression of sAPPa (*P*=.04) were also observed in the hippocampal samples of isolated APP/PS1 mice by Western blotting using 6E10 and densitometry analysis (Figure 4g-h). To determine whether decreased Aβ clearance is also involved in the exacerbated Aβ accumulation in isolated APP/PS1 mice, expression levels of NEP and IDE, 2 proteolytic enzymes that are mainly responsible for eliminating Aβ from the brain parenchyma (Fukami et al., 2002; Kasturirangan and Sierks, 2010), were compared between isolated and group APP/PS1 mice. We found that isolation housing triggered a decrease in NEP expression (*P*=.009) but did not alter IDE expression (*P*=.129) in the hippocampus (Figure 4e-f). Together, these results reveal that SI increased Aβ accumulation in the hippocampus of aged APP/PS1 mice, which was associated with upregulation of PS1 and downregulation of NEP.

### SI Decreased Hippocampal Volume and Synapse and Myelin-Related Proteins in Aged APP/PS1 Mice

**Figure 5.**
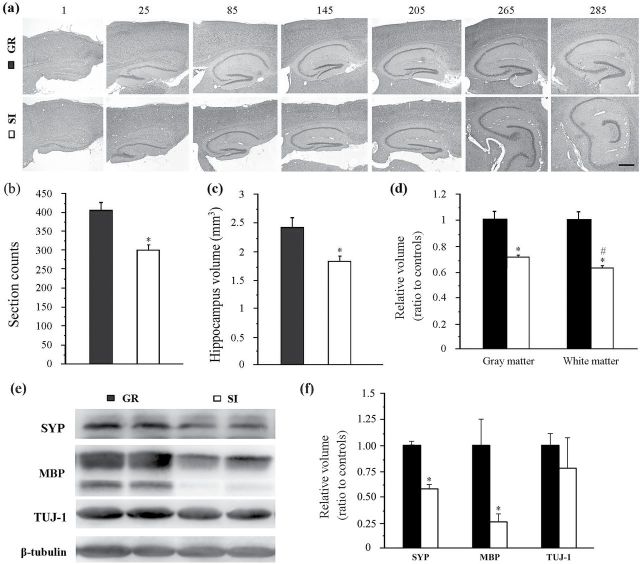
Analyses of hippocampal volume and expression levels of synapse, myelin, and neuron-related proteins. (a) Series of H&E-stained sagittal brain sections showing that the cross area of the dorsal hippocampus was smaller in isolated aged APP/PS1 mice than group controls. Note from the No. 265 section (about 1.68mm lateral to the mid-sagittal fissure), the intermediate region of the hippocampus, a transitional zone of the dorsal hippocampal region and ventral hippocampal region, was observed in isolated mice but was not present in group APP/PS1 mice until at No. 285 section. Scale bar=500 μm. (b) Counting the total number of sections that contain the dorsal hippocampus. (c) Quantification of the volume of the dorsal hippocampus. (d) Relative volume of the hippocampal gray matter and white matter between isolated aged APP/PS1 mice and group controls. (e-f) Western blotting and densitometry analysis of SYP MBP, and neuronal class III β-tubulin (TUJ-1). Data represent means ± SEM from 5 mice per group for pathological analyses and from 3 mice per group in 3 independent Western blotting analysis experiments. *P < .05, socially isolated (SI) aged APP/PS1 mice vs group (GR) controls. #*P* < .05, the relative volume of white matter vs the relative volume of gray matter.

The hippocampus is a vulnerable region to Aβ accumulation and is also sensitive to various AD-related risk factors such as hyperlipidemia, diabetes, hypertension, seizures, and stress (Dhikav and Anand, 2011). Indeed, hippocampal atrophy is a main neuropathological basis for irreversible impairment of spatial learning and memory in AD patients as well as in mouse AD models (Bird and Burgess, 2008). We examined whether isolation housing affects hippocampal size in aged APP/PS1 mice. As shown by HE-stained hippocampal serial sections, socially isolated APP/PS1 mice had a smaller dorsal hippocampus than group-housed controls (Figure 5a). Dorsal hippocampus section counts were reduced by 96 slices in isolated mice compared with group mice (*P*=.008) (Figure 5b). Volumetric analyses consistently showed a significant decrease in hippocampal volume of isolated APP/PS1 mice (*P*=0.034) (Figure 5c). Moreover, the white matter volume decreased more than that of the gray matter (*P*=.018) (Figure 5d), indicating that neurites undergo more prominent dystrophy or loss than neuronal cell bodies under AD-like pathology.

Synapses and oligodendrocytes are main components of the hippocampal white matter and are very vulnerable to the toxicity of Aβ peptides (Haroutunian et al., 2014). Previous studies have revealed that SI also impairs synapse and myelination plasticity in adult mice (Liu et al., 2012a; Pereda-Pérez et al., 2013). Thus, using Western-blot analyses, we determined whether SI affects synapse and myelin-related proteins by assessing SYP and MBP levels in the hippocampus. As expected, decreases in both SYP and MBP expression were observed in isolated APP/PS1 mice compared with group-housed controls (both *P*=.008) (Figure 5e-f). Additionally, immunostaining for PCNA and DCX revealed extremely low levels of generation or proliferation of immature neurons in the hippocampal dentate gyrus of both isolated and group aged APP/PS1 mice (data not shown), supporting the evidence that cell proliferation and neurogenesis are diminished in the aging mouse dentate gyrus (Kannangara et al., 2011). Taken together, these results suggest that exacerbated hippocampus atrophy, along with increased loss of synapse and myelin-related proteins, further contribute to the severe cognitive declines of isolated aged APP/PS1 mice.

### SI Increased Reactive Gliosis in the Hippocampus of Aged APP/PS1 Mice

**Figure 6.**
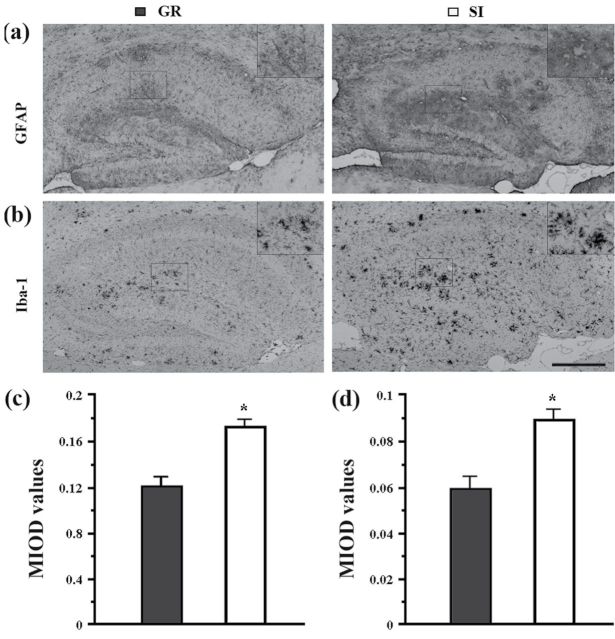
Analyses of reactive gliosis in the hippocampus. (a-b) Sagittal brain sections from isolated aged APP/PS1 mice and group controls were immunostained with GFAP and Iba-1 to show expression and distribution of astrocytes and microglia in the hippocampus, respectively. Scale bar=500 μm. (c-d) The quantification analysis of mean integrated optical density (MIOD) of GFAP (c) and Iba-1 (d). Data represent mean ± SEM from 5 mice per group. *P < .05, socially isolated (SI) aged APP/PS1 mice vs group (GR) controls.

Reactive astrocytes and microglia that surround amyloid plaques are a neuropathological hallmark of AD (Allen and Barres, 2009). Furthermore, there is a close association of reactive gliosis with the AD process (Avila-Muñoz and Arias, 2014). To investigate the effect of SI on reactive gliosis in aged APP/PS1 mice, hippocampal distribution of astrocytes and microglia was examined by immunostaining for GFAP and Iba-1, respectively. Isolated APP/PS1 mice showed increases in both GFAP and Iba-1 immunoreactivity surrounding plaques compared with group controls (Figure 6a-b). Quantitative analysis further confirmed that isolation housing increased GFAP or Iba-1-positive labeling intensity in the hippocampus of APP/PS1 mice (*P*=.001; *P*=.006, respectively) (Figure 6c-d), confirming its deteriorating role in Aβ-associated gliosis.

## Discussion

A social life where social creatures such as humans and rodents live in a community is vitally important to the overall health of the being. Correspondingly, scientific evidence reveals that SI is harmful to both physical and mental health (Seeman, 2000; Cacioppo et al., 2011; Steptoe et al., 2013). AD, a neurodegenerative disease with gradual declines in social, cognitive, and emotional functions, eventually separates the patients from social and family life (Luzzi et al., 2007; Weiss et al., 2008). However, the internal interaction between SI and AD still remains unclear. Previous experiments have shown that SI causes the early onset of AD-like phenotypes in young adult APP/PS1 mice (Hsiao et al., 2011, 2012). In this study, we demonstrate that aged APP/PS1 mice housed in a group exhibit more serious cognitive dysfunction after living in an isolated environment for 3 months. The pathological basis of the further cognitive deterioration includes more prominent brain Aβ deposit caused by increases in PS1 and decreases in NEP, which subsequently exacerbates hippocampal atrophy, synapse and myelin associated protein loss, and glial neuroinflammatory reactions. The finding suggests that AD patients’ conditions will worsen significantly, even in the late stages of the disease, when they become socially isolated. In contrast, interventions via social and family life might slow down the progression of AD or arrest further cognitive deterioration.

Although the etiology of AD remains unknown, a number of risk factors including hypertension, diabetes, hyperlipidemia, unhealthy lifestyle, and adverse social conditions are associated with the onset and progression of AD (Barnes and Yaffe, 2011; Jiang et al., 2013a; Barnard et al., 2014). An early epidemiological study showed that SI in older individuals increases the risk of developing AD (Wilson et al., 2007). Corresponding experimental studies reveal that SI exacerbates memory impairment in 3 month-old APP/PS1 mice in a time-dependent manner (Hsiao et al., 2011). The present results suggest that isolation housing has detrimental effects on cognitive declines during the late stages of AD. These data offer clear evidence that group or family life is not only beneficial to prevent the AD onset but can also to delay AD progression.

Apart from memory deficits, the severity of AD is also associated with impairment of exploratory behaviors, especially showing a loss of curiosity into novel surroundings or environment (Havins et al., 2012). In agreement with this view, isolated aged APP/PS1 mice showed low exploratory activity in the open field test compared with their group controls. In addition, anxiety or depression often occurs in the process of AD but has not been correlated with the severity of cognitive dysfunction (Panza et al., 2010; Maalouf et al., 2011). Previous studies have reported that rearing juvenile or adult rodents in SI environments elicits a variety of behavioral abnormalities, such as increased aggressiveness, anxiety, and hyperactivity (Weiss et al., 2004; Koike et al., 2009; Lukkes et al., 2009; Ouchi et al., 2013). In the present study, we found that isolated aged APP/PS1 mice showed no significant increases in water maze swimming speed and travelling speed in the Y maze, as well as the defecation number and grooming number in the open field, indicating that SI-induced hyperactivity and anxiety are not obvious in aged AD models. Together, the present behavioral data suggest that isolation housing selectively exacerbates cognitive impairment in the late stages of AD.

A considerable body of evidence indicates that an imbalance between Aβ production and clearance leads to Aβ accumulation, which plays a central role in the cognitive dysfunction and neurodegenerative changes that characterize AD (Mawuenyega et al., 2010; Holtzman et al., 2011). Along with more severe memory deficits, isolated aged APP/PS1 mice showed increased Aβ accumulation in the hippocampus accompanied with increased PS1 expression and decreased NEP expression. Early studies also revealed that 8-week isolated housing increases Aβ levels and γ-secretase activity in the hippocampus of 3-month-old APP/PS1 mice (Hsiao et al., 2012). Aside from SI, other manners of chronic stresses, such as chronic immobilization stress (Jeong et al., 2006) and mild unpredictable chronic stress (Cuadrado-Tejedor et al., 2012), exacerbate amyloid production in transgenic mouse models of AD. Furthermore, unpredictable chronic stress can also alter APP metabolism toward the amyloidogenic pathway in normal, middle-aged rats (Catania et al., 2009). These studies highlight that various stresses, including SI, stimulate increases in the amyloidogenic pathway. Further evidence indicates that increased glucocorticoids (Green et al., 2006; Li et al., 2010) and oxidative stress (Hsiao et al., 2012) contribute to SI-altered APP metabolism, although the underlying mechanisms warrant further investigation.

NEP and IDE are 2 major Aβ-degrading enzymes, with each playing an important role in maintaining homeostasis of Aβ in the normal brain (Kasturirangan and Sierks, 2010). NEP is typically expressed in axonal and synaptic membranes (Fukami et al., 2002) and is responsible for degrading monomeric and oligomeric forms of Aβ (El-Amouri et al., 2008). IDE is expressed predominantly in the cytosol of neurons but is also secreted from microglial cells and contributes to the degradation of polymeric and fibrillary forms of Aβ in the brain (Eckman and Eckman, 2005; Qiu and Folstein, 2006). Decreases in NEP mRNA, protein level, and activity are observed in the postmortem AD hippocampus (Yasojima et al., 2001; Caccamo et al., 2005; Wang et al., 2005), and NEP protein expression is inversely correlated with the severity of Aβ accumulation and cognitive impairment (Wang et al., 2010). In contrast, conflicting results exist for IDE in AD, and its protein expression seems not to be correlated with Aβ or clinical diagnosis (Zhao et al., 2007; Wang et al., 2010). Experimental studies also revealed that intracranial injection of AAV expressing NEP, but not IDE, reduces amyloid pathology in both the hippocampus and cortex of APP/PS1 mice (Carty et al., 2013). These findings suggest that NEP, rather than IDE, is a possible therapeutic target for Aβ degradation in AD. Consistent with these studies, the present results reveal that isolated housing decreases expression of NEP, but not IDE, in aged APP/PS1 mice. Taken together, isolated housing increases PS1 and decreases NEP expression, further exacerbating Aβ accumulation in the hippocampus of aged APP/PS1 mice.

Consistent with additional Aβ accumulation, the present results reveal that isolated housing decreases the hippocampal volume of APP/PS1 mice. The hippocampus is essential for declarative memory synthesis and hippocampal atrophy and is a core pathological substrate for cognitive impairment in AD patients (deToledo-Morrell et al., 2007; Barnes et al., 2009). Available evidence suggests that accumulation of Aβ, together with its subsequent pathological events, including formation of neurofibrillary tangles, disrupted mitochondrial energy metabolism, Ca^2+^ overload, activation of apoptotic pathways, oxidative stress, and glia-associated neuroinflammation, contribute to impairment of hippocampal structure and function (Dhikav and Anand, 2011). Besides Aβ and the examined secondary damage factors, several studies have revealed that SI itself has detrimental effects on hippocampal volume, neurogenesis, synapse plasticity, and axonal myelination (Stranahan et al., 2006, Djordjevic et al., 2009, 2012; Liu et al., 2012a; Pereda-Pérez et al., 2013). Indeed, the hippocampus has long been known as a target of stress hormones and is an especially plastic and vulnerable region of the brain (Joëls et al., 2003; Blank et al., 2004). The hippocampus expresses a high number of corticosteroid receptors and is responsive to circulating corticosteroids (Chaouloff and Groc, 2011). Elevated cortisol induces transcription of inhibitory glucocorticoid receptors, which are associated with depressed excitatory synaptic transmission and increased hippocampal atrophy (de Leon et al., 1998; Setiawan et al., 2007; Patel et al., 2008; Kamal et al., 2014). Furthermore, baseline plasma cortisol levels are elevated in the elderly compared with younger adults, which may identify cortisol as a key contributor to increased aging-associated hippocampal atrophy (Wright et al., 2005; McAuley et al., 2009). Isolated housing for 11 days does not change corticosterone in young (2-month-old) female C57BL/6J mice, but increases corticosterone significantly at the onset of the dark cycle in aged (17- to 18-month-old) mice (Kannangara et al., 2011). As mentioned above, glucocorticoids in turn increase Aβ production (Green et al., 2006; Li et al., 2010). Therefore, both SI induced Aβ-associated damage and SI itself are involved in the process of hippocampal atrophy in aged APP/PS1 mice.

In conclusion, the present results demonstrate that isolation housing exacerbates AD-like pathophysiology in aged APP/PS1 mice. It should be noted that the contribution of abnormal APP metabolism to SI-induced cognitive declines of aged mice has not been addressed in the present study. Furthermore, despite being one of the most widely used models, APP/PS1 mice cannot recapitulate fully all of the pathological features of AD. Thus, the internal interaction between SI and AD needs to be further confirmed by using multiple transgenic and/or natural models of AD. Clinical studies will also be necessary to investigate the potential benefits and mechanisms of group or family life in delay or counter the cognitive decline of AD patients. The potential findings will help establish useful nonpharmacological interventions, including lifestyle changes, to combat the devastating neurodegeneration.
